# HLA-class II haplotypes and Autism Spectrum Disorders

**DOI:** 10.1038/s41598-018-25974-9

**Published:** 2018-05-16

**Authors:** Meriem Bennabi, Alexandru Gaman, Richard Delorme, Wahid Boukouaci, Céline Manier, Isabelle Scheid, Nassima Si Mohammed, Djaouida Bengoufa, Dominique Charron, Rajagopal Krishnamoorthy, Marion Leboyer, Ryad Tamouza

**Affiliations:** 1 INSERM, U1160, Hôpital Saint Louis, Paris, France; 2 INSERM, U955, Psychiatrie Translationnelle, Créteil, France; 3 Fondation FondaMental, Créteil, France; 4 DHU Protect, AP-HP, Service de psychiatrie de l’enfant et de l’adolescent, Hôpital Robert Debré, Paris, France; 5 Département de génétique humaine et fonctions cognitives, Institut Pasteur, Paris, France; 6 Université Paris Diderot, Sorbonne Paris-Cité, Paris, France; 7 Laboratoire Jean Dausset and LabEx Transplantex, Hôpital Saint Louis, Paris, France; 8 DHU PePSY, AP-HP, Pôle de Psychiatrie, Hôpitaux Universitaires Henri Mondor, Créteil, France; 9 Université Paris-Est-Créteil, Faculté de médecine, Créteil, France

## Abstract

Infections and autoimmunity are associated with autism spectrum disorders (ASD), with both strongly influenced by the genetic regulation of the human leukocyte antigen (HLA) system. The relationship between ASD and the HLA genetic diversity requires further investigation. Using a case control design, the distribution of HLA class II-DRB1 and DQB1 alleles, genotypes and haplotypes were investigated in ASD patients, *versus* healthy controls (HC). ASD patients meeting DSM-IV TR criteria and HC (474 and 350 respectively) were genotyped at medium resolution using a Luminex-based SSO technology. Comparisons of genotypes, allele frequencies associated with a haplotype analysis were performed. Results indicate: (i) the HLA-*DRB1* *11-DQB1*07 haplotype was more prevalent in ASD patients, *versus* HC (*Pc* = 0.001), partially replicating previous data and possibly linking to gastro-intestinal (GI)-related pro-inflammatory processes, given that this haplotype associates with pediatric celiac disorders; (ii) the HLA-*DRB1* *17-DQB1*02 haplotype was higher in HC, *versus* ASD patients (*Pc* = 0.002), indicating that this is a protective haplotype. Using the Autism Diagnostic Interview to assess clinical dimensions, higher scores on social (*Pc* = 0.006) and non-verbal functioning (*Pc* = 0.004) associated with the *DRB1* *11 DQB1*07 haplotype. Our results support HLA involvement in ASD, with possible relevance to GI and gut-brain axis dysregulation.

## Introduction

Autism Spectrum Disorders (ASD) are heterogeneous neurodevelopmental disorders characterized by deficits in social communication as well as by repetitive patterns of behaviors and interests^1^. In recent decades, a striking increase in ASD prevalence has occurred, with 1/68 children diagnosed with ASD in the United States^2^. A number of factors are likely to have contributed to this rise, including improvements in diagnosis and epidemiological strategies as well as lifestyle changes, including exposure to environmental insults, new dietary habits and increased medication use^3^. Such data highlights the urgent need for a deeper understanding of the underlying pathophysiological processes in ASD, and thereby to innovative therapeutic strategies. Clarification of the underpinnings of gene and environment interactions represents a major theoretical and clinical target.

ASD is now widely accepted as being highly heritable^4^, with environmental risk factors interacting with genetic background. The development of ASD involves not only alterations in brain functioning and maturation, but also changes in immune processes. Alterations in immune functioning in ASD have been repeatedly demonstrated (for review, see^5^) showing, at least in subgroups of ASD patients: (i) a deleterious effect of prenatal or perinatal infectious pathogen exposure^6^; (ii) a pro-inflammatory state^7^ often concomitant with abnormal cell-mediated immunity^8^ or inflammatory-mediated gut dysbiosis^9,10^; (iii) a frequent autoimmune component observed in mothers of ASD off-spring as well as in ASD patients, with circulating anti-brain autoantibodies sometimes correlating with disease severity and/or behavior impairments^11–16^.

Such data clearly indicate a role for immune alterations in ASD, including interactions of the innate and adaptive immune systems. Previous research has reported relationships between the genetic diversity of loci encoding major immune molecules and ASD risk, including evidence of genetically-driven innate immune alterations in ASD^7,17–24^. Although the time scale for their interactions requires clarification, the co-existence of infections, inflammation and auto-immunity in ASD indicates that the foremost genetic susceptibility candidate(s) may be expected to lie in the vicinity of the highly polymorphic Human leukocyte antigen (HLA) super-locus^25^.

Hosted by the major histocompatibility complex (MHC) region on the short arm of the chromosome 6 (6p21.3–22.1), the HLA cluster is characterized by the highest polymorphism rate of the human genome^26^ (IMGT/HLA Database; http://www.ebi.ac.uk/imgt/hla). The cell surface HLA molecules, upon interactions with T cell receptors (TCRs), drive specific adaptive immune responses through antigen processing and presentation. While the HLA -A, -B and -C molecules encoded by the classical HLA class I gene cluster govern cellular immune processes, their class II counterparts (HLA-DRB1, -DQB1 and -DPB1, encoded by genes located in the HLA-class II sub-region) are crucial in mediating humoral immune responses. HLA molecules are also essential in a wide array of other physiological processes, including brain development and homeostasis^27,28^. Consequently, HLA genetic diversity has been intensively investigated in disease-association studies^29^, especially in regard to infectious, inflammatory and autoimmune disorders^25,29–31^.

In ASD, the influence of class I or II HLA polymorphisms has been explored, without providing any clear substantial evidence of a possible HLA-related pathway. This may be the consequence of the relatively small sample sizes used, the frequent use of non-molecular HLA genotyping techniques, and the scarcity of analysis at haplotype levels^32–46^. However, the polygenic contribution to the risk of several severe psychiatric disorders, including ASD, was confirmed in a meta-analysis of genome wide association studies (GWAS) where the MHC was the most significant shared risk loci^47^, reinforcing the notion that MHC-linked impairments in immune regulation may constitute a common risk factor across these disorders. However, despite the importance of these findings, it still requires clarification as to the precise involvement of any HLA locus in ASD, at least in part due to the imperfect coverage of HLA diversity in GWAS.

In order to provide additional information as to the role of HLA in ASD, we undertook a case-control study to more precisely explore the polymorphisms of the HLA class II loci, at allele, genotype and haplotype levels, using standardized molecular techniques.

## Results

From the total of 483 ASD and 352 HC subjects from the “PARIS study cohort”, HLA genotyping data was available only for 474 ASD and 350 HC subjects on which the statistical analysis were performed. The demographic and clinical characteristics of the study subjects are shown in Table 1.

**Table 1:** Demographic and clinical data of patients with Autism Spectrum Disorder (ASD) and healthy controls (HC).

Characteristics	ASD	HC
Number of subjects included in the study	474	350
Age at inclusion (median and range in years)	15.40 (3–66)	35.74 (4–64)
Male:Female ratio	371:103	181:169
IQ values: median and range	64 (7–146)	
DSM-IV TR Diagnosis		
Asperger	N = 58	
Typical autism	N = 324	
PDD-NOS	N = 92	
Autism diagnosis interview (ADI-R)	Values in median and range	
Social domain scores	22 (7–30)	
Non-verbal communication domain scores	11 (1–14)	
Verbal communication domain scores	17 (4–26)	
Repetitive behavior domain scores	6 (1–12)	

IQ: intellectual quotient; PDD-NOS: Pervasive developmental disorder not otherwise specified.

### HLA class II haplotype distribution in ASD subjects and healthy controls

Whilst no statistically significant differences at allele (Table 2) and genotype level were evident, the analysis of HLA-class II haplotype distribution showed that the HLA-*DRB1* *11-DQB1*07 haplotype was more prevalent in ASD patients, *versus* HC (14.5% vs 8.7% respectively; *Pc* = 0.001) (Table 3). This indicates a susceptibility status and replicates previous findings^36,48^. We also found that the frequency of the class II HLA-*DRB1* *17-DQB1*02 haplotype was significantly higher in HC, *versus* ASD patients (13.1% vs 9.2% respectively; *Pc* = 0.002), thereby indicative of a protective haplotype (Table 3). It is worthy to mention that the frequencies of HLA alleles constituting the above-mentioned haplotypes, albeit failing to reach significance, show the same trend of association i.e. HLA-*DRB1* *11 and HLA-DQB1*07 alleles more prevalent in ASD patients, *versus* HC (16% vs 9.1% and 22% vs 16.2% respectively) and HLA-*DRB1* *17 and HLA-DQB1*02 alleles more frequent in in HC, *versus* ASD patients (13.1% vs 9.2% and 25.5% vs 20.5% respectively) (Table 2).

**Table 2:** HLA-*DRB1* and HLA-*DQB1* allele frequencies in ASD patients and healthy controls.

HLA alleles	ASD N subjects = 474	HC N subjects = 350	p_c_value
	% (n alleles)	% (n alleles)	
*DRB1*			
DRB1*04	12.13 (115)	15.57 (109)	NS
DRB1*07	12.87 (122)	14.14 (99)	—
DRB1*13	13.19 (125)	13.57 (95)	—
DRB1*11	**16**.**03 (152)**	**9**.**14 (64)**	—
DRB1*15	13.61 (129)	10.29 (72)	—
DRB1*17	**9**.**28 (88)**	**13**.**14 (92)**	—
DRB1*01	8.97 (85)	10.57 (74)	—
DRB1*14	4.01 (38)	4 (28)	—
DRB1*16	3.48 (33)	2.57 (18)	—
DRB1*08	2.43 (23)	3.57 (25)	—
DRB1*12	1.69 (16)	1 (7)	—
DRB1*09	1.37 (13)	1 (7)	—
DRB1*10	0.42 (4)	1 (7)	—
DRB1*03	0.21 (2)	0.29 (2)	—
DRB1*18	0.32 (3)	0.14 (1)	—
*DQB1*			
DQB1*06	24.68 (234)	22 (154)	—
DQB1*02	**20**.**57 (195)**	**25**.**57 (179)**	—
DQB1*07	**22**.**05 (209)**	**16**.**29 (114)**	—
DQB1*05	18.46 (176)	18 (126)	—
DQB1*08	7.81 (74)	11 (80)	—
DQB1*04	2.74 (26)	3.86 (28)	—
DQB1*09	3.59 (34)	2.71 (19)	—

NS: statistically non significant.

**Table 3:** HLA class II haplotype frequencies among ASD patients and healthy controls.

HLA classe II haplotype (*DRB*1-*DQB1*)	ASD 455 subjects* % (n)	HC 337 subjects* % (n)	p_c_-value	OR (95%CI)
HLA-DRB1*11-DQB1*07	14.53 (69)	8.7 (30)	**0**,**00172**	**1**,**75 (1**,**24–2**,**47)**
HLA-DRB1*17-DQB1*02	9.22 (44)	13.14 (46)	**0**,**00245**	**0**,**75 (0**,**53–1**,**04)**
HLA-DRB1*04-DQBI*08	7.27 (35)	9.77 (34)	0,14624	NS
HLA-DRB1*04-DQB1*02	0.42 (2)	0.46 (2)	0,16188	—
HLA-DRB1*12-DQB1*07	1.29 (6)	0.76 (3)	0,20959	—
HLA-DRB1*10-DBQ1*05	0.56 (3)	0.91 (3)	0,22171	—
HLA-DRB1*15-DQB1*05	0.81 (4)	0 (0)	0,23538	—
HLA-DRB1*07-DQB1*08	0 (0)	0.78 (3)	0,32001	—
HLA-DRB1*15-DQB1*06	12.64 (60)	10.24 (36)	0,32672	—
HLA-DRB1*14-DQB1*05	4.48 (21)	3.51 (12)	0,32891	—
HLA-DRB1*13-DQB1*06	10.32 (49)	11.92 (42)	0,48848	—
HLA-DRB1*09-DQB1*09	0.98 (5)	0.76 (3)	0,55949	—
HLA-DRB1*07-DQB1*09	1.67 (8)	1.52 (5)	0,68934	—
HLA-DRB1*16-DQB1*05	3.36 (16)	2.59 (9)	0,68999	—
HLA-DRB1*04-DQB1*04	0.53 (3)	0.61 (2)	0,71389	—
HLA-DRB1*13-DQB1*07	1.40 (7)	1.37 (5)	0,72218	—
HLA-DRB1*04-DQB1*07	4.08 (19)	4.58 (16)	0,8153	—
HLA-DRB1*07-DQB1*02	10.64 (51)	10.84 (38)	0,82915	—
HLA-DRB1*01-DQB1*05	8.96 (43)	10.70 (38)	0,88199	—
HLA-DRB1*08-DQB1*04	2.10 (10)	2.90 (10)	0,92928	—

*Among the total of 474 ASD patients and 350 HC subjects, only reliable haplotype assignment by maximum likelihood estimates of haplotype probabilities are herein reported (for ASD: 455 and for HC: 337 subjects). NS: statistically non significant.

### Correlations between HLA class II risk haplotype and Autism Diagnostic Interview domains

**Figure 1:**
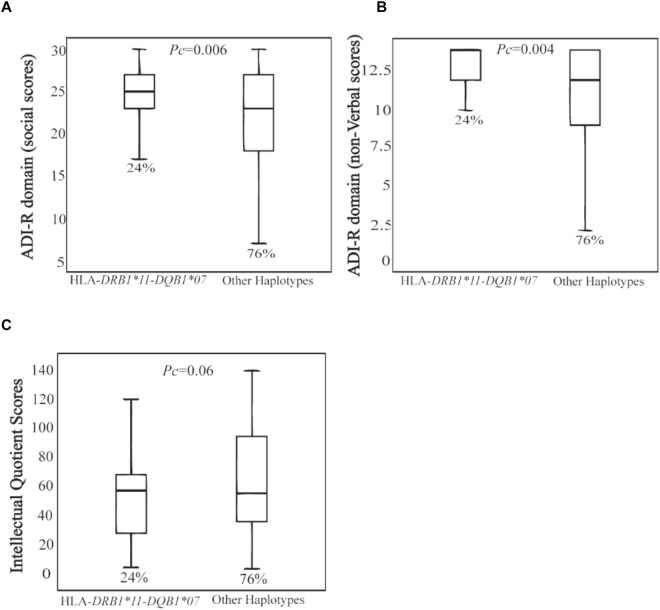
Correlations between HLA class II risk haplotype and ADI-R domain. The *DRB1* *11-DQB1*07 haplotype is more prevalent among patients with the higher scores on the ADI-R social domain (*Pc* = 0.006), ADI-R non-verbal domain (*Pc* = 0.004) (**A**,**B**) and among those having an IQ less 70 and considered as low functioning patients (*Pc* = 0.06) (**C**).

Importantly, analysis according to autistic symptomatology further confirmed the risk associated with the *DRB1* *11-DQB1*07 haplotype. This haplotype was more prevalent among ASD patients with the highest scores on the Autism Diagnostic Interview-Revised (ADI-R) social domain (*Pc* = 0.006) and ADI-R non-verbal domain (*Pc* = 0.004) as well as in ASD patients with an IQ below 70 or with low functioning ASD (*Pc* = 0.06) (Fig. 1).

## Discussion

Given the well-established functional role of the HLA system in innate and adaptive immune responses, and the frequently reported immune dysfunction in ASD, we have analyzed the distribution of the HLA-class II DRB1 and DQB1 alleles, genotypes and haplotypes in a sample of ASD patients, *versus* HC.

Findings indicate that the HLA-*DRB1* *11 -DQB1*07 haplotype is associated with ASD risk. This is in agreement with two previous studies reporting associations between a high frequency of the HLA-*DRB1* *11 allele and: (i) autism risk and concomitant decrease in CD4+ naive and increase in CD4+ memory T cells in Italian patients^36^; and (ii) autism risk and family history of autoimmune disorders in patients of Saudi Arabian origin^48^. The genetic distance between these two different cohorts, one from European descent and one from Middle East descent, and our cohort, may indicate a trans-ethnic validation of the HLA-*DRB1* *11 allele-related susceptibility status. Pathophysiologically, it is of note that the HLA DQB1*07 haplotype is well proven to associate with celiac disease (CD), an immune enteropathy sharing several gastro-intestinal (GI) abnormalities with ASD. Indeed, triggered by gluten-derived antigenic peptides presented by specific HLA-class II molecules in genetically predisposed individuals, CD is characterized by intestinal inflammation leading to digestive manifestations, similar to those encountered by ASD patients^49,50^. ASD patients often present with GI symptoms such as diarrhea, abdominal pain or bloating often correlated with increased ASD severity^51^. Accounting for around 40% of the disease heritability, the genetic component of CD is mainly due to HLA-DQA and DQB genes encoding the DQ2 and DQ8 molecules. In addition, several studies including a recent large survey investigating the influence of HLA haplotypes on CD development in those at risk (CD relatives or CD like symptoms), demonstrate that the most frequent CD-associated haplotype in DQ2 and DQ8 negative is represented by the HLA-DQ7 haplotype (corresponding to DQ loci encoding the DQ7 specificity in linkage disequilibrium with HLA-DRB1*11/12 alleles)^52^. Although the subject of some controversy^53,54^, the link between ASD and CD has been recently demonstrated by a retrospective study showing a higher incidence of biologically proven CD in ASD children, *versus* an ethnically matched general pediatric population^55^. Given that between 23–70% of ASD patients have GI symptoms^56,57^, it is not unlikely that the susceptibility status conferred by the HLA-*DRB1* *11-DQB1*07 haplotype reflects overlapping pathophysiological processes between CD and a subset of ASD patients. A limitation of the present study is the absence of any information concerning GI symptoms in ASD patients. Indeed, in the absence of data on CD in this ASD cohort it is not possible to assess at present if the risk haplotype is associated with ASD *per se* or as a consequence of CD.

The second major finding regards the protective status conferred by the HLA-*DRB1* *17-DQB1*02 sub-haplotype. This may be understood in the context of HLA ancestral haplotype (AH) characteristics. Indeed, the HLA-*DRB1* *17-DQB1*02 sub-haplotype constitutes the 5′ part of the 8.1 AH “autoimmune haplotype” (A*01-B*08-DRB1*03(17)-DQB1*02), which is the most frequent AH among those of European descent and the most strongly associated HLA haplotype with several dysimmune conditions, including chronic inflammation and autoimmunity^58,59^. In healthy individuals, the 8.1AH is characterized by increased circulating levels of the pro-inflammatory cytokine, tumor necrosis factor alpha, and an increase in the Th2/Th1 ratio, favoring HLA-mediated humoral responses^58^. Given the pro-inflammatory burden associated with this haplotype, it can be hypothesized that individuals with this haplotype can mount more efficient anti-infectious responses. This may be relevant to the role of infectious prenatal or perinatal events in the etiology of ASD^6^.

Another possible explanation of the observed protective status conferred by the HLA-*DRB1* *17-DQB1*02 haplotype may be linked to the involvement of the HLA system in wider physiological processes, including synaptic pruning. This may have parallels to a recent study demonstrating the implication of a neuro-developmental, genetically-driven complement (C4) pathway in the etiology of schizophrenia^60^. The latter study shows strong relationships among elevated C4 copy number variations, the presence of the human endogenous retrovirus K (HERV-K) and increased expression of C4A molecules, with both related to excessive synaptic pruning during specific developmental windows. Notably, 8.1 AH, which was previously associated with protection against schizophrenia^61^, structurally lacks the HERV-K^62^, which may thereby counteract C4A overexpression and reduce the risk of schizophrenia. Therefore, in the present study it cannot be excluded that the 8.1 AH may mediate the observed protective status, through an as yet to be defined, pruning processes during neuro-developmental windows.

Overall, our findings may better reconcile previous data and indicate that the clarification of HLA genetic diversity will be important to understanding the dysimmunity repeatedly observed in ASD. This will be enhanced by recent technological approaches, such as next generation sequencing, allowing analysis at the sequence level of the entire MHC region after haplotype-based selection.

## Material and Methods

### Subjects and clinical assessments

Subjects meeting DSM-IV-TR criteria for ASD were enrolled under the cadre of the Paris Autism Research International Sibpair study (PARIS study), and carried out in specialized clinical neuropsychiatric centers established in France and Sweden^63^. Patients were assessed with the Autism Diagnostic Interview-Revised (ADI-R)^64^ and with the Autism Diagnostic Observation Scale (ADOS)^65^ and included only after a thorough clinical evaluation with psychiatric and neuropsychological examination, standard karyotyping, and fragile-X testing, as well as brain imaging and EEG. Assessment of intellectual ability was carried out with an age-appropriate Wechsler scale (WPPSI, Wechsler Preschool and Primary Scale of Intelligence; WISC, Wechsler Intelligence Scale for Children; or WASI, Wechsler Abbreviated Scale of Intelligence). For the most severe and/or non-verbal patients, the Raven’s Standard Progressive Matrices were used to measure nonverbal IQ (NVIQ) and the Peabody Picture Vocabulary Test (PPVT-4th edition) to measure receptive vocabulary (RV). The healthy control (HC) group consists of unrelated healthy individuals, with no personal or familial psychiatric disorders^66,67^.

Written informed consent was obtained from all participants, including from caregivers/guardians on behalf of children included in the study, and the documents recorded and stored in each participating center (Paris and Gothenburg). The study was approved by the local Institutional Review Board (IRB) i.e. the “Comités de Protection des Personnes (CPP) Île-de-France, Hôpital Pitié-Salpêtrière 75013 Paris” for France and the “Sahlgrenska Academy Ethics committee, University of Gothenburg” for Sweden. The entire research and all methods were performed in accordance with the relevant guidelines and regulations.

### HLA genotyping

Genomic DNA was extracted from EDTA-treated peripheral blood samples using the Nucleon BACC3 kit (GE HealthCare, Chalfont St Giles, UK). HLA two digits intermediate resolution typing for HLA-DRB1 and HLA–DQB1 alleles was performed using PCR-sequence specific oligonucleotide (SSO) Luminex LABTYPE^®^SSO kits designed to recognize all the broad specificities based on the sequence databases from IMGT/HLA Database (database version 3.17.0) (http://www.ebi.ac.uk/ipd/imgt/hla/probe.html). The Luminex 100 flow analyzer identified HLA alleles *via* HLA visual 1.0 software, by referring to HLA typing template data for the studied loci as provided by the manufacturer (OneLambda, Inc. CA). The detected alleles are assigned into their corresponding serological specificities with consequent HLA typing results as serological equivalents. HLA-DQ2 to -DQ9 will be the corresponding serological specificities for HLA-DQB1 alleles while those corresponding to HLA-DRB1*01:01 to –DRB1*10:01 alleles will be HLA-DR1 to -DR18.

### Statistical analysis

Quantitative variables are described with mean, standard deviation, median and inter-quartile range. Qualitative variables are described with counts and percentages. Distributions of quantitative variables were compared according to the levels of a group variable with the Student’s t-test or the ANOVA test when data are normally distributed, otherwise with the Mann-Whitney test or the Kruskal-Wallis test. Distributions of qualitative variables were compared according to the levels of a group variable with the chi-square test. The risks were measured with Odds ratio and 95% confidence interval. Corrections for multiple tests were used with the Benjamini-Hochberg method^68^. Alpha-risk of the significance was set to 5%.

Haplotype frequencies were calculated with maximum likelihood estimates of haplotype probabilities for all the subjects and for each subset defined by the levels of a group variable. Only autosomal loci are considered. Statistical scores were performed to evaluate the association of a trait with haplotypes, when linkage phase is unknown and diploid marker phenotypes are observed among unrelated subjects. Only haplotypes with a minimum sample count of 5 were included in the analysis. Alpha-risk of the significance was set to 5%. Corrections for multiple tests were used with the Benjamini-Hochberg method.

The Kruskal-Wallis or Mann–Whitney tests were used for non-parametric analysis [distribution of HLA haplotypes according to intellectual quotient (IQ) values, as well as social and non-verbal functioning]. Linear regression analyses were performed to examine the relationship between IQ, social and non-verbal functioning, with HLA haplotypes and diagnosis as the predictive variables. By default, calculations assume that a two-tailed statistical test was used at a confidence level of 95%.

All analyses were performed with R version 3.2.0 (2015-04-16). The package haplo.stats (version 1.7.1) was used for the haplotype study^69^.
